# Eight generations of native seed cultivation reduces plant fitness relative to the wild progenitor population

**DOI:** 10.1111/eva.13243

**Published:** 2021-05-04

**Authors:** Riley Pizza, Erin Espeland, Julie Etterson

**Affiliations:** ^1^ University of Minnesota Duluth Duluth MN USA; ^2^ United States Department of Agriculture, ARS Sidney MT USA

**Keywords:** fitness, native plant cultivation, native seed farm, restoration, seed increase, unconscious selection

## Abstract

Native seed for restoration is in high demand, but widespread habitat degradation will likely prevent enough seed from being sustainably harvested from wild populations to meet this need. While propagation of native species has emerged in recent decades to address this resource gap, few studies have tested whether the processes of sampling from wild populations, followed by generations of farm cultivation, reduce plant fitness tolerance to stress over time. To test this, we grew the eighth generation of farm‐propagated *Clarkia pulchella* Pursh (Onagraceae) alongside seeds from two of the three original wild source populations that established the native seed farm. To detect differences in stress tolerance, half of plants were subjected to a low‐water treatment in the greenhouse. At the outset, farmed seeds were 4.1% heavier and had 4% greater germination compared to wild‐collected seed. At maturity, farmed plants were 22% taller and had 20% larger stigmatic surfaces, even after accounting for differences in initial seed size. Importantly, the mortality of farmed plants was extremely high (75%), especially in the low‐water treatment (80%). Moreover, farmed plants under the high‐water treatment had 90% lower relative fitness than wild plants due to the 1.3 times greater weekly mortality and a 3‐fold reduction in flowering likelihood. Together, these data suggest that bottlenecks during initial sampling and/or unconscious selection during propagation severely reduced genetic diversity and promoted inbreeding. This may undermine restoration success, especially under stressful conditions. These results indicate that more data must be collected on the effects of cultivation to determine whether it is a suitable source of restoration seed.

## 1 INTRODUCTION

Anthropogenic activity, including climate change, has resulted in large‐scale ecosystem destruction and degradation (IPCC, 2018; Segan et al., 2016), reducing productivity across 20% of the planets’ vegetated surface (UNEP, United Nations Environment Programme, 2012). In response, the United Nations has called for an aggressive effort to restore natural landscapes and the ecosystem services they provide, designating 2021–2030 as “the decade of restoration” (UNEP, 2019). During this time period, UNEP aims to restore 350 million hectares of native habitat, requiring an unprecedented supply of native seed and plant restoration material. Given the critical nature of this endeavor, it is imperative we have access to a sufficient supply of restoration materials that will establish plant populations that will persist into the future.

Historically, many large‐scale restoration projects have relied upon affordable and commercially available native plant cultivars (Baer et al., 2014; Gustafson et al., 2005; reviewed in Leger & Baughman, 2015; United States Department of Agriculture, Natural Resources Conservation Service(n.d)). However, it has been shown that cultivar plantings may fail to restore ecosystem function (reviewed in Kettenring et al., 2014; Lesica & Allendorf, 1999; White, 2016). For example, a recent meta‐analysis showed that almost 25% of studied non‐local cultivars differed for phenotypic traits, such as flower color, which may decrease their pollinator attraction or suitability (Kramer et al., 2019). Another study showed that native bees visited native plants more often than their “nativar” (native cultivar; White, 2016), which could undermine the long‐term stability of the restored population. Although restoring ecosystem function is difficult in the best restoration scenarios (Herrick et al., 2006), the use of native plant material is more likely to support higher trophic levels and maintain other important environmental attributes, such as the nutrient composition of the soil (reviewed in Kettenring et al., 2014; Reynolds et al., 2012). In light of these facts, progressive governments, such as in Germany, have passed federal regulations requiring the exclusive use of local native plant material for habitat restoration (Kiehl et al., 2014; Mainz & Weiden, 2019) and others have called for higher quality control (Ladouceur et al., 2018; Schmidt et al., 2019).

Even if native seed sources are used for restoration, their origin may influence their likelihood of success at the restoration site. It is recommended that restoration plant material originates from large, genetically diverse wild populations, either local or intentionally selected from elsewhere to bolster genetic diversity (reviewed in Falk et al., 2013; Hufford & Mazer, 2003; Kettenring et al., 2014; Menges, 2008; but see Zeldin et al., 2020) or accommodate climate change (Broadhurst et al., 2008; Breed et al., 2013; Prober et al., 2015; reviewed in Bucharova et al., 2019). There is increasing concern, however, that repeated harvesting from wild populations may threaten their demographic stability (Broadhurst, Driver et al., 2015; Menges et al., 2004), especially for uncommon and endangered species (Guerrant, 1992). Therefore, alternative seed sources are needed, especially in light of the rising demand for native plant material (UNEP, 2019).

**FIGURE 1:**
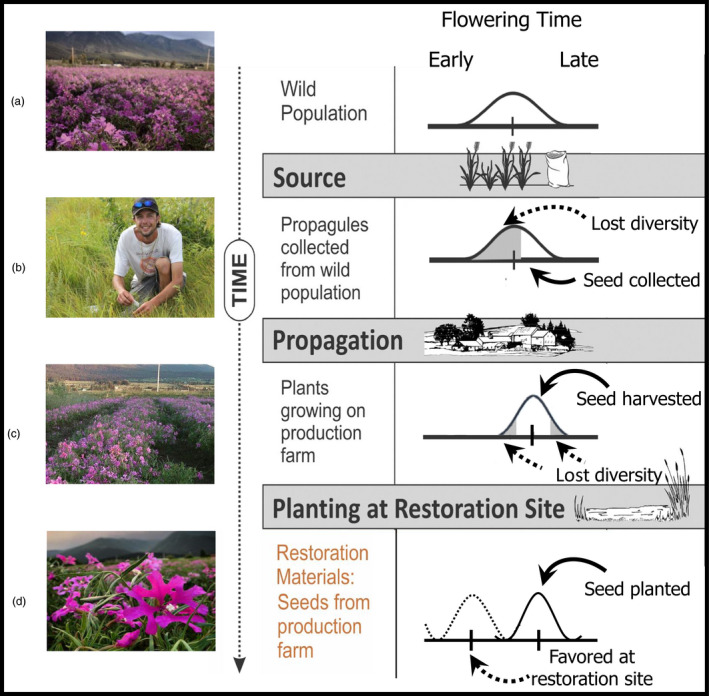
Hypothesized reductions in genetic diversity and unconscious selection in native seeds cultivated for restoration. A wild population (a) may have a large diversity of flowering times. Collection of seeds from this population late in their flowering season may reduce the genetic diversity of flowering time in the sampled population (b). This would result in unconscious selection for a plant population that now flowers later than the sampled wild population. When harvested again at peak seed production, further reductions in flowering time diversity are possible (c). Combined reductions in genetic diversity and unconscious selection over several generations of farm propagation may result in plants that are maladapted to the restoration site (d). Figure adapted from Espeland et al. (2017), photos courtesy of Native Ideals seed farm

Wild populations can be protected by harvesting a sustainable amount of seed and then increasing seed number on a production farm to meet demand (Broadhurst, Hopley et al., 2015; Kiehl et al., 2014; Oldfield, 2019; Shaw et al., 2005; Tischew et al., 2011; White et al., 2018). Although this process reduces demographic pressure on wild populations, it may have unintended consequences that ultimately reduce plant fitness at the restoration site (Espeland et al., 2017). First, the timing and frequency of wild seed collections may influence the extent and nature of genetic diversity in the farmed population (Espeland et al., 2017). Second, cultivated native plants may be watered, fertilized, and/or subjected to mechanical harvesting, each of which could inadvertently exert selection and elicit evolutionary change (Chivers et al., 2016; Espeland et al., 2017). Finally, once established, farmed populations may be repeatedly harvested (perennials) or replanted (annuals) for one or many sequential generations which could produce genetic bottlenecks and promote inbreeding (Espeland et al., 2017; Tischew et al., 2011). To illustrate these points, imagine a wild population with a range of seed maturation times (Figure 1a) If that population is sampled once at the peak of seed maturation, genotypes that flowered earlier and had already dispersed seed, or those that flowered later and are still maturing their seed, may be excluded (Figure 1b). Genetic losses may be exacerbated over time under cultivation as similar processes of sampling and unconscious selection (sensu Gross et al., 2014) continue to modify the genetic integrity of the farmed population. Ultimately, this could result in phenotypic shifts, such as later flowering time relative to the original wild population (Figure 1c). After only a few generations, the cultivated population may have been unconsciously selected to flower later than the original wild population and/or have reduced variation in flowering time (Figure 1d). Evolutionary changes may ultimately affect plant fitness at the restoration site (Figure 1d).

There are numerous traits that may be inadvertently selected in a production farm that are similar to “domestication traits” such as the loss of seed dormancy, increased seed size, uniform phenology, and a greater reliance on self‐fertilization (Chivers et al., 2016; Espeland et al., 2017; Gross & Olsen, 2010; Poelman et al., 2019). Larger seeds, for example, may be unconsciously selected for if they are more likely to germinate under deeper burial depths (Baskin & Baskin, 1998), as would be the case for mechanically planted seeds. Greater self‐fertilization may be unconsciously selected for if pollinator reliability is low (Bontrager et al., 2019). Another trait, rapid vertical growth, may be unconsciously selected for if it results in greater fecundity (reviewed in Tardieu, 2011) although it may reduce drought tolerance (e.g., Koziol et al., 2012). While these traits may be advantageous on the farm, they are unlikely to be useful in the wild (Espeland et al., 2017; but see Mercer et al., 2007). If domestication traits are favored during cultivation on native seed farms, restoration material may have maladaptive traits when they are planted into restoration sites.

To ensure the genetic integrity of farmed populations through the production process, some seed certification agencies regulate the number and size of sampled wild populations and/or limit the number of generations that farmed populations can be repeatedly harvested before being refreshed with new collections from the wild (Mainz & Weiden, 2019; Nagel et al., 2019; Pedrini & Dixon, 2020; Tischew et al., 2011). However, this level of seed quality control is extremely regional, and enforcement is often minimal (Pedrini et al., 2020). Current seed certification practices in the United States rely primarily on measures of “pure live seed” (Elias et al., 2006; Frischie et al., 2020; Stevens & Meyer, 1990; United States Department of Agriculture, Natural Resources Conservation Service, 2004). However, since it is still legal to sell uncertified seed (De Vitis et al., 2017; Mainz & Widen, 2019), producers must choose to follow less expensive conventional practices with higher risks of genetic erosion, or voluntary incur increased production costs to follow recommendations to decrease the risk of genetic erosion. Many seed producers choose the former, reasoning that it is better for practitioners to use lower‐quality native seed than equally priced cultivars (Broadhurst, Driver et al., 2015; Smith et al., 2007).

Although there is a dearth of information on the prevalence of genetic erosion during crop cultivation (reviewed in Govindaraj et al., 2015) and phenotypic shifts due to unconscious selection (Mercer et al., 2007; Rosenthal & Dirzo, 1997; reviewed in Keneni et al., 2012; Flint et al., 2019), only two studies have tested whether these processes occur during native seed production and obtained mixed results (Dyer et al., 2016; Nagel et al., 2019). Moreover, no studies have investigated whether changes that have occurred during cultivation also impact a plant's responses to stress, even though generational differences due to unconscious selection may be more apparent under these conditions (Armbruster & Reed, 2005). Based on the limited number of studies and the lack of congruency in the results, more research is necessary to determine the impact of cultivation on the fitness of restoration seeds. Without more conclusive evidence of genetic problems arising from the cultivation of native species, it is difficult to justify the added expense of stringent seed certification regulations aimed at preserving genetic diversity (Smith et al., 2007).

To contribute to the body of information on the extent and importance of sampling effects and unconscious selection on native seed farms, we compared *Clarkia pulchella* Pursh (Onagraceae) plants that had been cultivated for eight generations at Native Ideals seed farm (Arlee, MT) to plants from the original wild populations from which the farm population was established. Plants were raised in a greenhouse and subjected to two watering levels. We address the following questions: (1) Have farmed plant traits diverged from their wild progenitors in response to cultivation? (2) Do farm and wild plants respond differently to water stress?

## 2 METHODS

### 2.1 Study species

*Clarkia pulchella* Pursh (Onagraceae) is a commonly used species in habitat restoration in the western United States (McManamen et al., 2018). It has a western montane distribution from southern British Columbia to California (Lewis & Lewis, 1955) where it occurs on dry rocky slopes (Lewis, 1953). *C*. *pulchella* is a self‐compatible winter annual with herkogamy and protandry that promotes outcrossing (Lewis, 1953). In nature, plants can achieve a maximum height of 50 cm and produce many purple flowers that are typically pollinated by solitary bees (Lewis, 1953). *C*. *pulchella* does not have specialized mechanisms for seed dispersal, and the seeds are not known to persist in the seed bank (Newman & Pilson, 1997).

### 2.2 Seed sources

We obtained eighth‐generation cultivated seeds from a Native Ideals seed farm in Arlee, Mt (47.152713 N, 114.036317 W). Native Ideals Seed Farm established its farmed population in 2009 by pooling seed across three natural populations (47.025072 N, 114.440356 W; 46.895219 N, 113.9453673 W; and 46.878649 N, 113.985910 W), mixing seed with sand, and hand‐sowing in mid‐October onto a field pretreated with roundup. The following year, plants were treated once with a fungicide to reduce rust fungus but otherwise were not watered or fertilized. In early July, plant stalks were harvested with a manual swather and dried, the fruits were removed, and seeds were separated with a fanning mill. The harvested seeds were stored at ~10°C in cardboard containers until fall planting. This process was repeated for eight generations at which point we obtained the cultivated seeds.

For comparison to the cultivated population, we collected seed from two of the three original wild populations that established the Native Ideals Seed Farm population. The third population had been extirpated (46.878649 N, 113.985910 W), possibly due to roadside herbicide. We also attempted to sample this population from the soil seed bank by collecting 10 cm deep surface samples from three 1 × 1 m plots. However, no seedlings emerged from the seed bank samples from the extirpated population, in contrast to the other two extant populations where numerous germinants were recovered. Consequently, it was not possible to include this source population in our experiments. For the two extant populations, seed was bulk collected twice during a two‐week period in July during the peak of seed maturation; one to three fruits were collected from maternal plants that were separated by at least 1.5 m.

### 2.3 Germination and growth conditions

To establish the greenhouse experiment, 750 wild‐collected and 750 farmed seeds were individually weighed, positioned on dimpled blotter paper in petri dishes (eight petri dishes per seed source, 100 seeds in each dish), watered, and incubated in a growth chamber at 24°C day/18°C night temperature cycle with a 12‐h photoperiod. Germination date, which we denoted by a crack in the seed coat and a visible radicle, was monitored daily under a dissecting microscope for 30 days. Once cotyledons emerged from the seed coat, seedlings were transplanted into seed starting trays and maintained in the incubator. Then, surviving seedlings (*n* = 738) were individually transplanted into RC10U cone‐tainers (Stuewe and sons) with a 3:1 proMix HP soil (Gardeners Supply Company): sand (Nurserymen's Preferred) mixture and arranged in a randomized block design (32 blocks) in the greenhouse (July–October 2018). Blocks were randomized weekly across the greenhouse benches. Plants were watered daily for 50 days, fertilized bimonthly (5 ml Peters 20/20/20 NPK dissolved in 4 L of water; ICL Specialty Fertilizers), and grown under natural light until the first of September when a 12‐h light cycle commenced. To reduce aphid damage, infested plants were sprayed weekly with a soap solution (15 ml of soap—Dr. Bronners Pure‐castile liquid soap—diluted with 1 L of water). To reduce thrip damage, all plants were sprayed with insecticide (Conserve SC, Corteva Agriscience) once per week.

### 2.4 Watering treatment

After 50 days in the greenhouse, planting blocks were randomly assigned to a high‐ or low‐water treatment. Plants in the high‐water treatment were watered regularly (every 1–2 days). Plants in the low‐water treatment were watered when at least half of the plants were visibly wilted (2–3 days). Measurements of leaf water content were used to determine the effect of the watering treatment. At three time points during the experiment, young leaves at least 10 mm long were excised, weighed in microcentrifuge tubes, and placed uncapped in a drying oven at 65°C for 24 h. The tubes were reweighed, and water content was calculated as follows:

$$\text{water content}=\frac{\text{wet weight}‐\text{dry weight}}{\text{wet weight}}\times 100.$$

### 2.5 Growth and phenology measurements

Plant height and the length and width of the longest leaf were recorded weekly from the time of transplantation until senescence. The date of first flowering and the number of flowers open per day was also recorded. After observing initial differences in stigma size between seed sources, corolla diameter and stigma diameter were quantified by measuring the first two fully open flowers on each plant. Due to overall reductions in flowering, extensive thrip damage to flowers and seed pods, we were unable to produce crosses and collect seed from these plants. At the end of the experiment (after over half the plants began senescing), the roots of each plant were extracted from the soil. After the aboveground biomass was removed at the soil line, the cone‐tainers were soaked in warm water for 30 min to separate soil and roots. The contents of the cone‐tainer were sifted through two sieves 3.35 and 0.710 mm mesh opening, and larger pieces of soil (woodchips, perlite, etc.) were hand‐removed. The roots were placed into coin envelopes, dried, and weighed.

### 2.6 Data analysis

Seed mass data were analyzed with an ANOVA (JMP pro 13 software, SAS Institute, 2019). One extreme seed mass outlier (5.7 SD > mean seed mass) was removed in this, and in all subsequent analyses, although other measurement data from this individual were retained. The probability of germination was analyzed with a generalized linear model (GLM) assuming a binomial distribution and using a logit link. The number of days to germination was analyzed using a model with block (i.e., petri dish) nested within seed source as a fixed effect, as this GLM platform did not permit nested random effects. Coefficients of variation (CV) were compared using Levene's test to determine whether seed sources differed with respect to trait variation. Block was significant in three analyses (see Table S2 for results).

To account for environmental maternal effects that likely differed between wild‐collected and farm‐harvest seeds, we used seed mass as a covariate in all other trait analyses, which been commonly done previously (Bischoff & Müller‐Schärer, 2010; Huber et al., 1996; Picó et al., 2004; Weiner et al., 1997). All models also shared the following fixed effects: seed source, watering treatment, and the two‐way and three‐way interactions between these factors. The two‐way interaction between seed source and seed mass and the three‐way interaction between seed source, seed mass, and watering treatment were only retained in the model if they were significant. Block was nested within watering treatment and considered a random effect.

Flowering proportion and survival to week 21 were analyzed using a GLM using a binomial distribution and a logit link. Block was again treated as a fixed effect due to limitations of the statistical platform. The number of days to flowering, flowering duration, maximum number of flowers on a plant on any given day, total number of flowers, height at flowering, flower diameter, stigma diameter, and root mass were analyzed with a mixed model. To account for allometry in floral size traits, corolla diameter was used as a covariate in the analysis of stigma diameter (see Table S2). Data were transformed to meet analysis assumptions, although all graphs show untransformed data to facilitate interpretation. Each week of water content measurements was analyzed separately with a mixed model. Three extreme water content outliers were removed in the third measurement to improve data structure (Grubbs, 1950; see Table S3). Both a linear fit model and polynomial fit model (second degree) were run to test the relationship between the third water content measurement and plant height, but only the polynomial was reported as it was a better fit. Traits that were measured multiple times (i.e., height and length of the longest leaf) were analyzed with repeated measures ANCOVA. Mortality date was analyzed using a proportional hazards survivorship model.

**FIGURE 2:**
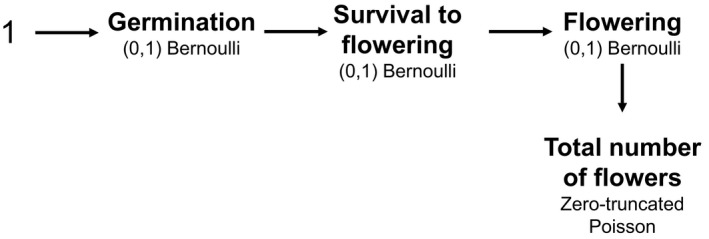
Path diagram illustrating aster model nodes. Each node is associated with a variable, each variable has a specified distribution, and the root node is a constant 1. Arrows and orders of variables indicate which variables are conditional based on the predecessor variable

To obtain a comprehensive measurement of the relative fitness of wild and farmed populations under contrasting water regimes, we used an aster model (R Core Team, aster version 0.9.1.1, Geyer et al., 2007; Shaw et al., 2008). This model allowed us to determine the absolute fitness of each of the four groups considering both categorical variables (germination, survival, and flowering) and a continuous variable (total number of flowers), while also accounting for conditional nature of life‐history traits (Figure 2). Once absolute fitness means were produced for each of the groups, relative fitness was calculated by dividing each group mean by the mean of the group with the highest fitness. While seed mass was included in this model, block was not included to prevent overparameterization.

## 3 RESULTS

After eight generations of cultivation, the farmed *Clarkia pulchella* population differed significantly from the wild seed source for over half of the measured morphological and phenological traits. Water availability also affected over half of the traits, sometimes as a main effect, sometimes as an interaction with seed source, or a complex interaction between the seed source, watering treatment, and seed mass.

### 3.1 Germination

**FIGURE 3:**
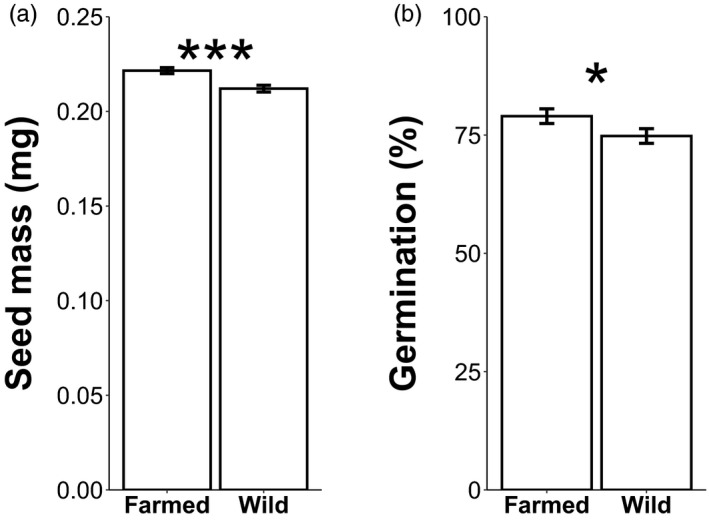
Seed mass and germination rate of wild and farmed plants. Least squares means (± standard error) for (a) seed mass (*n* = 1499) and (b) germination proportion (*n* = 1148) for wild and farmed *C*. *pulchella* seeds grown on petri dishes in an incubator. For χ^2^ statistics, see Table 1. *p < 0.05. **p < 0.01. ***p < 0.001

Farmed seeds were 4.1% heavier than wild seeds (Table 1; Figure 3a; Table S2) and had significantly higher germination rates (79% vs. 75%, respectively, Figure 3b; Table S2). Although the average time to germination did not differ between populations, germination phenology of the farmed population was significantly less variable than the wild population (Table S1).

**TABLE 1:** ANCOVA (*F*) and generalized linear model (χ^2^) test statistics

Factor	Seed source (SS)		Watering treatment (WT)		SS × WT		Seed mass (SM)		SM × WT		SM × SS		SS × WT × SM	
	df	*F*/(χ^2^)	df	*F*/(χ^2^)	df	*F*/(χ^2^)	df	*F*/(χ^2^)	df	*F*/(χ^2^)	df	*F*/(χ^2^)	df	*F*/(χ^2^)
Seed mass	1	18.09***	–	–	–	–	–	–	–	–	–	–	–	–
Germination proportion	1	3.73*	–	–	–	–	1	4.59*	–	–	–	–	–	–
Days to germination	1, 14	2.69	–	–	–	–	1, 894	0.72	–	–	–	–	–	–
Flowering proportion	1	65.66***	1	0.05	1	9.92**	1	0.82	1	4.94*	–	–	–	–
Days to first flower	1, 133	2.71	1, 71	0.15	1, 133	0.27	1, 131	2.94^†^	1, 131	3.07^†^	–	–	–	–
Maximum # of flowers	1, 134	0.04	1, 54	1.53	1, 134	0.21	1, 134	0.42	1, 134	0.32	–	–	–	–
Flowering duration	1, 132	0.20	1, 114	4.62**	1, 132	0.77	1, 131	1.53	1, 131	2.59	1, 131	2.60	1, 131	3.01^†^
Total # of flowers	1, 100	0.67	1, 85	5.66*	1, 100	1.89	1, 101	0.02	1, 101	0.85	–	–	–	–
Height at flowering	1, 131	10.80**	1, 131	5.02*	1, 131	2.89^†^	1, 130	0.90	1, 130	1.02	1, 130	0.70	1, 130	4.81*
Stigma diameter	1, 86	16.84***	1, 72	0.07	1, 86	0.63	1, 87	0.66	1, 87	3.28^†^	1, 87	0.05	1, 86	3.97*
Survival likelihood	1	9.00**	1	15.69***	1	0.17	1	0.11	1	0.42	–	–	–	–

Note

Morphological and life‐history traits were measured on two seed sources of *Clarkia pulchella*, a population that had been cultivated for eight generations on a native seed farm and the wild progenitor population. Plants were reared in the greenhouse and subjected to low‐ or high‐water treatment.

^†^ *p* < 0.10.

* *p* < 0.05.

** *p* < 0.01.

*** *p* < 0.001.

### 3.2 Flowering

**FIGURE 4:**
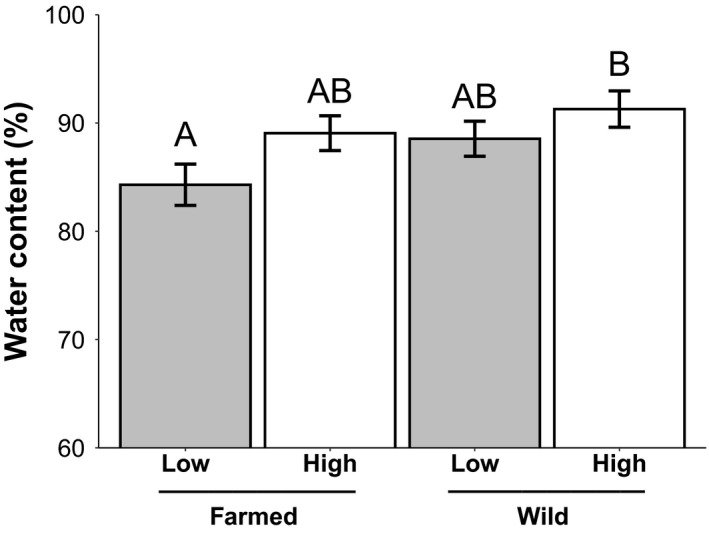
Water content in wild and farmed plants under different watering treatments at week 15. Least squares means (± standard error) for the third trial of % water content (*n* = 87) for wild and farmed *C*. *pulchella* plants grown in high‐water (white) or low‐water (gray) treatment in the greenhouse. Three outliers were removed per Grubbs outlier test (Grubbs, 1950) and although the y axis starts at 60, it encompasses all data points. For ANCOVA statistics, see Table S3

Anthesis began 10 weeks after germination and continued until week 21. The likelihood of flowering differed between seed sources both as a main effect and as an interaction between seed source and watering treatment (Table 1). Overall, plants from the wild population were more likely to flower than those from the farm; only 15% of farmed plants had bloomed by the end of the experiment compared to 60% of wild plants. Additionally, plants from different seed sources responded to the watering treatment in different ways; farmed plants had a low likelihood of flowering regardless of the watering treatment whereas wild plants were twice as likely to bloom in the high‐watered treatment relative to the low‐water treatment (Figure 4). Although flowering phenology did not differ significantly between the seed sources, there was a marginally significant interaction between seed mass and watering treatment (Table 1). Dissection of the interaction showed that while seed mass did not affect germination phenology under the well‐watered treatment, plants that germinated from smaller seeds were more likely to flower under the low‐water treatment.

The maximum number of flowers on a plant on any given day did not differ between seed sources or watering treatment, nor did the total number of flowers (Table 1). However, the duration of flowering—the number of days between the first and last flower—was affected by the watering treatment; plants flowered, on average, ten fewer days under the low‐water treatment compared to plants in high‐water treatment. There was also a marginally significant three‐way interaction between watering treatment, seed source, and seed mass (Table 1). Dissecting the three‐way interaction, seed mass did not affect the duration of flowering for wild plants in either watering treatment. For farmed plants, in contrast, seed mass had opposite effects on flowering depending upon the watering treatment; well‐watered farmed plants that germinated from smaller seeds flowered for more days. However, under the low‐water treatment, plants that germinated from larger seeds ultimately flowered for more days. There was no difference in the total number of flowers produced between the seed sources, although plants in the low‐water treatment produced significantly fewer flowers than those in the high‐water treatment (Table 1).

**FIGURE 5:**
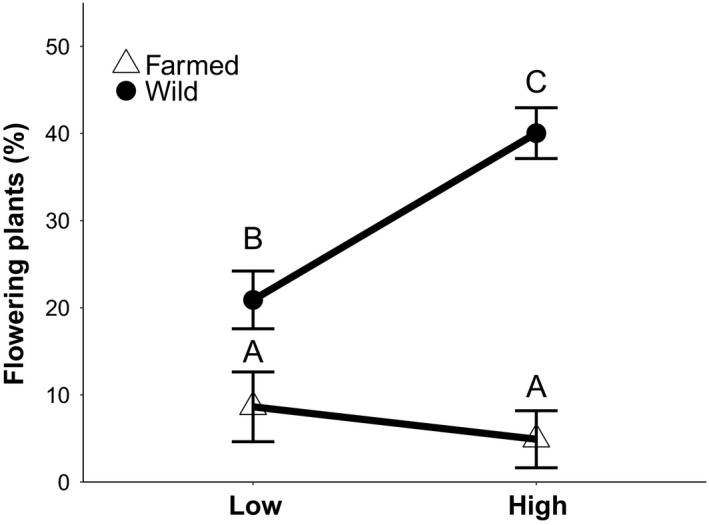
Flowering proportion for wild and farmed plants under different watering treatments. Interaction plot showing least squares means (± standard error) of the proportion of farmed (triangles) and wild (circles) flowering *C*. *pulchella* plants under high‐water (*n* = 380) and low‐water (*n* = 358) treatment. For GLM statistics, see Table 1

Plant height at anthesis differed significantly between seed sources and watering treatments (Table 1). While farmed plants were generally taller than wild plants, only farmed plants in the high watering treatment were significantly taller than wild plants at both watering levels (Figure 5a). There was also a significant three‐way interaction between seed source, watering treatment, and seed mass (Table 1); farmed plants that germinated from smaller seeds were ultimately taller at flowering in the low‐water treatment and shorter in high‐water treatment, whereas the opposite was true for wild plants. Farmed plants also had significantly larger stigma diameters compared to wild plants regardless of watering treatment (Figure 5b; Table S2). There was also a marginally significant two‐way interaction between seed mass and watering treatment and a significant three‐way interaction between seed source, seed mass, and watering treatment. Dissection of the three‐way interaction shows that the stigma diameter of wild plants was not influenced by seed mass in either watering treatment, whereas the stigma diameter of farmed plants was heavily influenced by seed mass: Plants germinating from heavier seeds had larger stigma diameters in the high‐water treatment and smaller diameters in the low‐water treatment. There was no significant difference in flower diameter between seed sources or watering treatments (Table S3).

### 3.3 Plant structure

**FIGURE 6:**
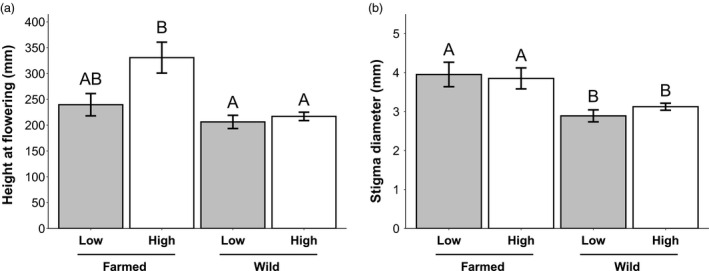
Height at flowering and stigma diameter of wild and farmed plants under different watering treatments. Least squares means (± standard error) for (a) height at flowering (*n* = 142) and (b) stigma diameter (*n* = 97) for wild and farmed *C*. *pulchella* plants grown in high‐water (white) or low‐water (gray) conditions in the greenhouse. For ANCOVA statistics, see Table 1

Leaf water content was high and did not differ between populations or treatments until week 15, at which time watering treatments differed significantly although the seed sources did not (Table S3). Specifically, farmed plants in the low‐water treatment had 7% lower water content than wild plants in the high‐water treatment (Figure 6). There was no correlation between plant height and water content (*F*_1,32_ = 1.57, *p* = 0.22, *r* ^2^ = 0.09). Root mass did not differ between seed sources, although it was 30% lower in the low‐water treatment (Table S3).

### 3.4 Growth rate

**FIGURE 7:**
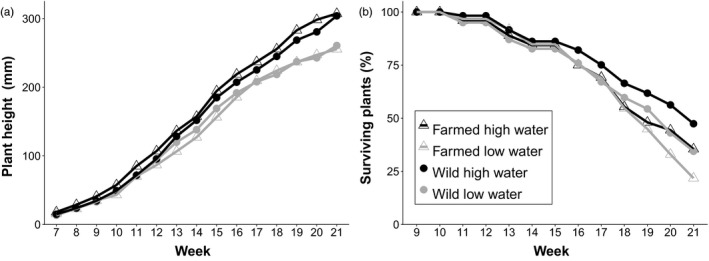
Growth and survival of wild and farmed plants under different watering treatments. Least squares means (± standard error) for (a) plant height (*n* = 737) and (b) % of plants alive each week (*n* = 737) for wild (circle) and farmed (triangle) *C*. *pulchella* plants grown in high‐water (black) or low‐water (gray) conditions in the greenhouse. Repeated drought conditions began at week 9. For ANCOVA statistics, see Table 2

The overall average growth rate in terms of plant height did not differ between seed sources but was significantly slower in the low‐water treatment compared the high‐water treatment (Table 2). The growth rate of the seed sources also differed over time (Time × Seed Source) and according to the watering treatment (Time × Watering Treatment). Wild plants in the low‐water treatment were approximately 11% shorter per week than their high‐water counterparts, and low‐watered farmed plants were 18% shorter per week than high‐watered plants (Figure 7a). Leaf growth also differed significantly between seed sources and watering treatments (Table 2). Farmed plants had, on average, 9.5% longer leaves each week compared to wild plants, and plants in the high‐water treatment had leaves ~13% longer each week than plants in the low‐water treatment. Although leaves were longer each subsequent week, there were no significant interactions between time and any other factor in the analysis (Table 2).

**TABLE 2:** Repeated measures ANCOVA tests (*F*)

	Plant height		Longest leaf	
	df	*F*	df	*F*
Within weeks				
Seed source (SS)	1, 239	0.09	1, 126	4.98*
Watering treatment (WT)	1, 239	8.09***	1, 126	6.57**
Seed mass (SM)	1, 239	0.73	1, 126	0.02
SM × WT	1, 239	0.18	1, 126	0.10
SS × WT	1, 239	1.2	1, 126	0.09
Block	2, 239	3.22*	2, 126	1.19
Between weeks				
Time	14, 226	3.30***	14, 113	1.74^†^
Time × SS	14, 226	2.07*	14, 113	1.09
Time × WT	14, 226	2.14**	14, 113	0.67
Time × SM	14, 226	0.73	14, 113	0.40
Time × SM × WT	14, 226	0.98	14, 113	0.57
Time × SS × WT	14, 226	1.21	14,113	0.95

Note

Differences within each week of measurement, and over the course of the study (between weeks) for measurements taken on wild and farmed populations of *C*. *pulchella* subjected to low‐water or high‐water treatment in the greenhouse.

^†^ *p* < 0.10.

* *p* < 0.05.

** *p* < 0.01.

*** *p* < 0.001.

### 3.5 Survival

**FIGURE 8:**
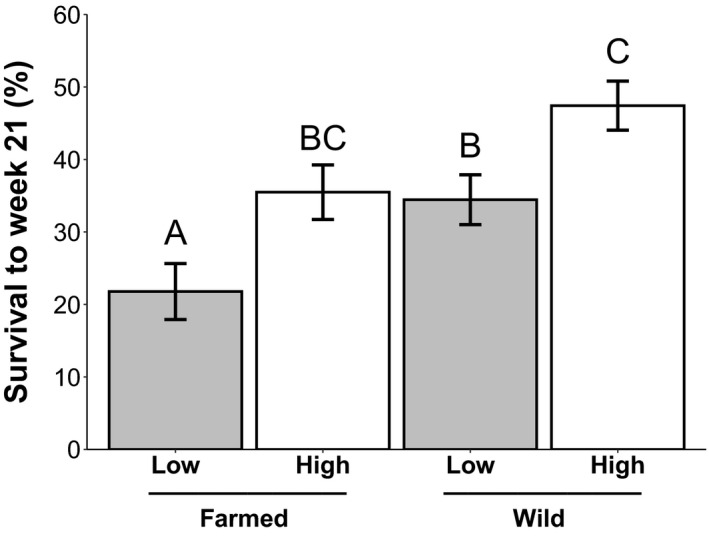
Lifetime survival of wild and farmed plants under different watering treatments. Least squares means (± standard error) of the % of plants that flowered and survived to the end of the experiment (week 21) for wild (*n* = 402) and farmed (*n* = 335) *C*. *pulchella* plants grown in well‐watered (white) or low‐water (gray) conditions in the greenhouse. For ANOVA statistics, see Table S3

The risk of mortality differed between the seed sources and watering treatments (Table 1). Farmed plants were 1.3 times more likely to die in any given week compared to wild plants. In addition, plants in the low‐water treatment had a 1.4 times greater risk of mortality than plants in the high‐water treatment (Figure 7b). By the end of the experiment, survival differed between seed sources and watering treatments. Within each watering treatment, the survival rate of wild plants was ~10% higher than farmed plants. Additionally, both seed sources experienced a similar decrease in survival under the low‐water treatment (~20%), although farmed plants under low‐water treatment had significantly lower survival than all other groups (Figure 8; Table S3).

### 3.6 Relative fitness

**FIGURE 9:**
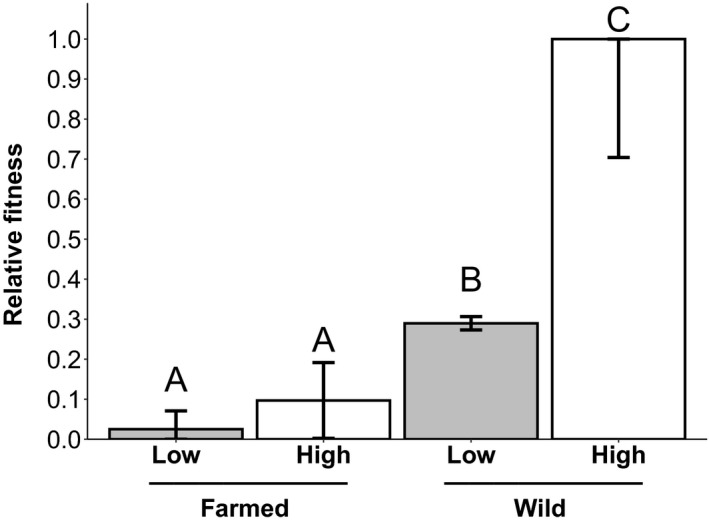
Predicted relative fitness measures for wild and farmed plants under differing watering treatments. Estimated means (± standard error) for wild and farmed *C*. *pulchella* (*n* = 1471) plants grown in high‐water (white) or low‐water (gray) treatment in the greenhouse. Relative fitness measurements were estimated using an aster model

The relative fitness of the wild plants was significantly greater than that of farmed plants (Figure 9) when jointly considering germination, flowering, and the total number of flowers produced in an aster analysis (Geyer et al., 2007; *z* = 3.77, *p* < 0.001). The response to the watering treatment was the same for both seed sources; plants under high‐water treatment had significantly higher fitness than those under low‐water treatment (*z* = 2.02, *p* = 0.04). Overall, regardless of watering treatment, wild plants had significantly greater fitness than farmed plants. Seed mass was not a significant predictor of fitness (*z* = 0.83, *p* = 0.41).

## 4 DISCUSSION

As the global demand for habitat restoration continues to rise, restoration practitioners must obtain a native seed supply without compromising the demographic stability of wild populations through overharvesting (Broadhurst et al., 2008; Meissen et al., 2017). While production on native seed farms has been considered a viable alternative source of plant material to support the ambitious goals of the “decade of restoration,” few studies have tested whether genetic degradation or other evolutionary changes are occurring during the cultivation process that may undermine restoration success (Dyer et al., 2016; Nagel et al., 2019). Here, we show that after only eight generations of cultivation, propagated *Clarkia pulchella* differed significantly from the wild progenitor populations in several functional traits that result in lower fitness, especially under abiotic stress.

Two previous studies have addressed the same fundamental issues and obtained mixed results. In a common garden comparison of the parental wild and F1 cultivated generation of two perennial grass species, Dyer et al. (2016) found significant changes in cultivated plants after just one generation. In contrast, Nagel et al. (2019) found few changes when comparing cultivated F1–F5 generations of five species with diverse life histories (parental generation not included). Interestingly, the species that was most similar to *C*. *pulchella* in this study, the short‐lived predominantly selfing species *Medicago lupulina*, showed pronounced changes. Specifically, there was a nine‐fold decrease of flowering between F1 and F4. The authors concluded that harvesting plants in late August unintentionally removed small, early flowering plants from the cultivated population, resulting in generations F2–F4 consisting only of large, late‐flowering plants. To the best of our knowledge, no study to‐date has reported reductions in fitness comparable to our results. It may therefore be hypothesized that such a dramatic reduction in fitness must be uncommon in the native seed industry. However, given the lack of data in this field, the existence of this study warrants further exploration to determine the extent to which these results may be observed at other native seed farms.

Importantly, the declines in fitness that we observed at later developmental stages (i.e., flowering) were not evident in measurements of germination. This is problematic, as current seed standards and certification practices in the United States and elsewhere (Pedrini & Dixon, 2020), are largely based on quantification of “pure live seed” (Elias et al., 2006; Frischie et al., 2020; United States Department of Agriculture, Natural Resources Conservation Service, 2004). The assumption underlying this practice is that high germination rates are correlated with high fitness at later developmental stages (e.g., Pywell et al., 2003). However, this may not always be the case, especially if the cause of low fitness is inbreeding depression; a large meta‐analysis comparing the effects of inbreeding depression on seed germination found that in over half of studies, inbred seeds had equal, or higher, germination rates compared to outbred seeds (Baskin & Baskin, 2015). Inbreeding could happen readily on native seed farms if the same base population is repeatedly harvested and replanted for several generations (Bison et al., 2004; Charlesworth & Charlesworth, 1999; Johnston, 1992; Koelewijn, 1998; Montalvo, 1994; Reed & Frankham, 2003), as is the case in this study. Consequently, the failure of plants to become established at the restoration site might be falsely attributed to factors that commonly undermine restoration success such as invasion (reviewed in Kettenring & Adams, 2011), poor weather, or disturbance (Norland et al., 2018) rather than low seed quality. Thus, it is important that later stages of plant development, especially fecundity, are periodically assessed to confirm the quality of seeds produced on native seed farms.

One of the most surprising results in study was the low overall likelihood of flowering (~50%). This may be related to thrip damage that was widespread, especially on flower buds, and is known to prevent flowering in some species (e.g., Wien & Roesingh, 1980). The growth conditions and photoperiod for this species may have also been suboptimal given that *C*. *pulchella* is a winter annual that germinates in the fall and overwinters under snow cover (Engelen‐Eigles & Erwin, 1997; Lewis & Lewis, 1955; Visperas et al., 1987), although other researchers have grown wild‐collected *C*. *pulchella* without vernalization and in unnatural photoperiods without such extreme reductions in flowering (Bontrager & Angert, 2019; Bontrager, personal communication). Although we only measured reproductive potential, the number of farmed plants that never flowered support that reproductive success would be significantly lower for farmed plants. If our results are not unique and, more importantly, if cultivation causes similar fitness declines in other annual species, restoration goals may be undermined.

### 4.1 Unconscious selection for domestication traits

One concern about native seed production is that traits similar to domesticated species may evolve as a consequence of unconscious selection (Dyer et al., 2016; Espeland et al., 2017). We found evidence that supports this possibility at all developmental stages of *C*. *pulchella*. Farmed seeds were heavier than their wild counterparts, as is commonly observed between cultivars and their wild relatives (Gross & Olsen, 2010). Larger seed size may evolve if producers assume that small seeds have lower viability and cull them during the seed‐cleaning process (Basey et al., 2015). This would be an unfounded assumption; a large meta‐analysis showed that inbred seeds were often equal in size, or larger, than wild seeds (Baskin & Baskin, 2015). Mechanical planting may also favor larger seeds if they have higher germination success at the greater burial depths (Baskin & Baskin, 1998; Kluyver et al., 2013). However, a shift to a larger seed size is concerning for restoration material that may be introduced to highly disturbed sites, where traits associated with smaller seeds increase survival (Kulpa & Leger, 2013). Moreover, since farmed seeds had a significantly smaller variation in seed mass, and farmed seeds were significantly larger, cultivated populations may lack the phenotypes most likely to succeed at degraded restoration sites. This is further supported in our data, as farmed plants performed poorly under low‐water conditions as may be expected in a cleared restoration site.

Another striking difference between wild and farmed plants was that the latter had significantly larger stigmas, which has been positively associated with pollen reception (Eckert, 2000). Increased pollen reception may be especially advantageous in environments, such as a monospecific native seed farm, where the plant is unlikely to receive heterospecific pollen (Waser, 1978; but see Montgomery & Rathcke, 2012). A larger stigmatic surface may also reduce herkogamy and subsequently increase selfing, which may be beneficial in environments where pollinators are rare (Eckert et al., 2006; Opedal et al., 2016). For example, a study done on *Mimulus luteus* populations found that plants with less herkogamy produced more seeds than those with greater herkogamy when pollinators were absent (Carvallo & Mendel, 2010). Another study found that wild populations of *C*. *pulchella* under climate stress had greater selfing rates when pollinator reliability was low (Bontrager et al., 2019). However, a shift to higher selfing rates may also lead to inbreeding depression (Charlesworth & Charlesworth, 1999; Herlihy & Eckert, 2004; Lynch & Walsh, 1998) and explain the lower fitness of farmed plants (Takebayashi & Delph, 2000).

Differences between wild and farmed populations could also have been influenced by environmental carry‐over effects that are especially pronounced in early life‐history traits (Bischoff & Müller‐Schärer, 2010; Montalvo, 1994). Although we used seed mass as a covariate as has been done previously (e.g., Bischoff et al., 2006; Picó et al., 2004; Weiner et al., 1977), it was not significant in most of our analyses. Some of the differences may also be attributed to the absence of one of wild populations in this study. However, it is unlikely that the missing wild population could explain the low fitness of the farmed population. Even if the missing wild population was highly inbred (i.e., Buza et al., 2000; Koelewijn, 1998), eight generations of mating with the other two wild populations on the farm would have relieved inbreeding depression (Frankham, 2015, 2016; Hedrick, 1994). Therefore, although we cannot ensure that the missing wild population does not account for some of our results, it is unlikely to explain them all.

### 4.2 Response to water stress

Finally, our study was unique in that it was the first to intentionally test whether cultivated native plants differ in their stress response relative to wild plants. Since several differences were observed only when a watering treatment was implemented, we recommend that future studies test whether farmed plants have reduced tolerance other stresses such as herbivory (Welter & Steggall, 1993) or interspecific competition (Weiner et al., 2010). Either of these may be unconsciously selected against during cultivation, especially if tolerance results in a trade‐off with growth or fecundity (reviewed in Muller‐Landau, 2010). In our study, we hypothesized that farmed plants would have a reduced tolerance to the low‐water treatment due to a trade‐off with rapid growth rate (e.g., Kozoil et al., 2012). Although farmed plants were taller in both watering treatments, there was no correlation between plant height and water content. Finally, some genetic differences between plant populations, especially those caused by inbreeding, are not visible until plants experience specific stressors (reviewed in Armbruster & Reed, 2005). Therefore, there may be differences between wild and farmed plants that remain undetected.

## 5 CONCLUSIONS

Given the limited number of studies that have directly tested the effects of cultivation on farm‐produced native seed (Dyer et al., 2016; Nagel et al., 2019), we do not know the extent of genetic erosion within commercially produced native seed for restoration. Further study is warranted, including impacts at the restoration site. If additional evidence suggests that this is a common problem, regulatory measures may be necessary. Smith et al. (2007) found that even when the genetic risks of certain cultivation practices are understood, native seed farmers fear that additional costs will incentivize customers to buy non‐local, low‐quality, and cheaper seed. Thus, until there are certification processes in place that indicate seed quality (e.g., Frischie et al., 2020; Pedrini & Dixon, 2020) that not only include the percentage of pure live seed but also measures of reproductive fitness, economic drivers during seed production and at the marketplace are likely to prevail.

## CONFLICT OF INTEREST

The authors have no conflicts of interest to disclose.

## Supporting information

Table S1

Table S2

Table S3

## Data Availability

Data for this manuscript are openly available at the Dryad Digital Repository: https://doi.org/10.5061/dryad.1c59zw3v8 (Pizza et al., 2021).
