# Supplementary material for: Eight generations of native seed cultivation reduces plant fitness relative to the wild progenitor population
Source: Evol Appl. 2021 May 4;14(7):1816–29. doi: 10.1111/eva.13243 (PMC8288025; doi:10.1111/eva.13243)
Supplement: Supplementary file 1 — Table S1 [file EVA-14-1816-s001.docx]

TABLE S1: Coefficient of Variation (CV) calculations.

**Notes:** Differences in trait variation for measurements taken on wild and farmed populations of *C. pulchella* subjected to low-water or high-water treatment in the greenhouse. Lavene tests produced p-values for these values and are indicated with asterisks. This test was only used to compare differences between seed sources within each watering treatment and were not run on groups with less than 10 measurements.
