# Supplementary material for: Eight generations of native seed cultivation reduces plant fitness relative to the wild progenitor population
Source: Evol Appl. 2021 May 4;14(7):1816–29. doi: 10.1111/eva.13243 (PMC8288025; doi:10.1111/eva.13243)
Supplement: Supplementary file 2 — Table S2 [file EVA-14-1816-s002.docx]

TABLE S2: Significant Block and Additional Covariates from ANCOVA (F) and GLM (χ2) Test Statistics**.**

|  | **Block** | | **Corolla Diameter** | |
| --- | --- | --- | --- | --- |
| **Factor** | *df* | (*χ^2^*) | *df* | *F /* *(*χ^2^) |
| Germination proportion | 1 | **67.92***** | --- | **---** |
| Days to germination | 1 | **6.34**** | --- | **---** |
| Stigma diameter | --- | --- | 1, 86 | **5.45*** |
| † p < 0.10; * p < 0.05; ** p < 0.01; *** p < 0.001 | | | | |
| Table S3: |  |  |  |  |

Notes: Morphological and life history traits were measured on two seed sources of *Clarkia pulchella*, a population that had been cultivated for eight generations on a native seed farm and the wild progenitor population. Plants were reared in the greenhouse and subjected to low or high-water treatment.
