# Supplementary material for: Eight generations of native seed cultivation reduces plant fitness relative to the wild progenitor population
Source: Evol Appl. 2021 May 4;14(7):1816–29. doi: 10.1111/eva.13243 (PMC8288025; doi:10.1111/eva.13243)
Supplement: Supplementary file 3 — Table S3 [file EVA-14-1816-s003.docx]

Table S3: Additional ANCOVA (F) and generalized linear model (χ^2^) test statistics**.**

|  | **Seed source**  **(SS)** | | **Watering**  **Treatment**  **(WT)** | | **SS**  **×**  **WT** | | **Seed mass**  **(SM)** | | **SM**  **×**  **WT** | |  |  |  |  |
| --- | --- | --- | --- | --- | --- | --- | --- | --- | --- | --- | --- | --- | --- | --- |
| **Factor** | *df* | *F /* (*χ^2^*) | *df* | *F /* *(*χ^2^) | *df* | *F /* (χ^2^) | *df* | *F /* (χ^2^) | *df* | *F /* (χ^2^) |  |  |  |  |
| Water content wk 11 | 1, 52 | 0.95 | 1, 53 | 1.47 | 1, 52 | 2.23 | 1, 52 | 1.46 | 1, 52 | 0.31 |  |  |  |  |
| Water content wk 13 | 1, 52 | 1.91 | 1, 52 | 0.01 | 1, 52 | 0.87 | 1, 53 | 0.08 | 1, 53 | 1.02 |  |  |  |  |
| Water content wk 15 | 1, 79 | 2.16 | 1, 75 | **8.12**** | 1, 79 | 0.63 | 1, 80 | 0.01 | 1, 80 | 0.01 |  |  |  |  |
| Flower diameter | 1, 58 | 0.30 | 1, 3 | 0.71 | 1, 58 | 0.18 | 1, 48 | 0.18 | 1, 48 | 1.49 |  |  |  |  |
| Root mass | 1, 279 | 1.13 | 1, 279 | **17.78***** | 1, 279 | 0.68 | 1, 279 | 1.67 | 1, 279 | 0.02 |  |  |  |  |
| \| Survival to wk 21 \| 1, 279 \| 1.13 \| 1, 279 \| **17.78***** \| 1, 279 \| 0.68 \| 1, 27 \| 1.67 \| 1, 279 \| 0.02 \| **---** \| **---** \| **---** \| **---** \| \| --- \| --- \| --- \| --- \| --- \| --- \| --- \| --- \| --- \| --- \| --- \| --- \| --- \| --- \| --- \| | 1 | **12.61***** | 1 | **13.62***** | 1 | 0.32 | 1 | 0.03 | 1 | 0.07 |  |  |  |  |
| † p < 0.10; * p < 0.05; ** p < 0.01; *** p < 0.001 | | | | | | | | | | |  |  |  |  |
| Notes: Morphological and life history traits were measured on two seed sources of *Clarkia pulchella*, a population that had been cultivated for eight generations on a native seed farm and the wild progenitor population. Plants were reared in the greenhouse and subjected to low or high-water treatment and measured each week (wk). | | | | | | | | | | |  |  |  |  |
|  |  |  |  |  |  |  |  |  |  |  |  |  |  |  |
|  |  |  |  |  |  |  |  |  |  |  |  |  |  |  |
|  | | | | | | | | | | | |  |  |  |
